# Supplementary material for: Serum 25-hydroxyvitamin D Concentration Significantly Decreases in Patients with COVID-19 Pneumonia during the First 48 Hours after Hospital Admission
Source: Nutrients. 2022 Jun 7;14(12):2362. doi: 10.3390/nu14122362 (PMC9228147; doi:10.3390/nu14122362)
Supplement: Supplementary file 1 [file nutrients-14-02362-s001.zip › Nutrients_supplementary_figures.pdf]

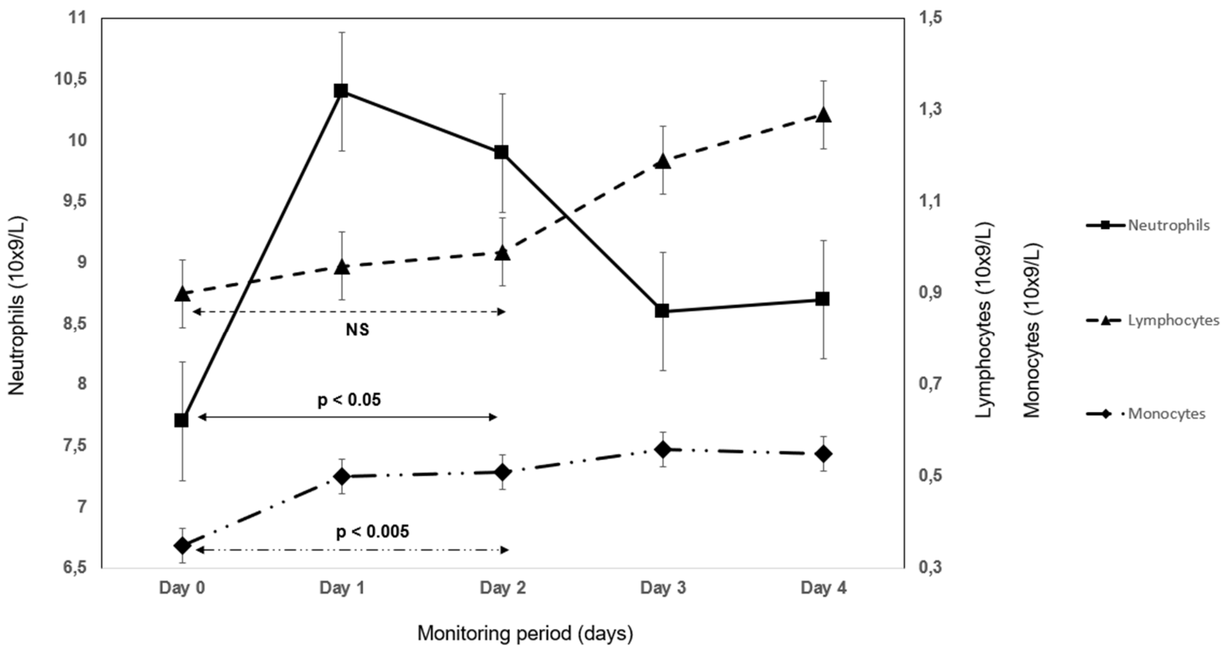

Figure S3: The changes of mean values of the neutrophils, lymphocytes, and monocytes during the monitoring period. P values of the change during the first 48 hours are displayed.

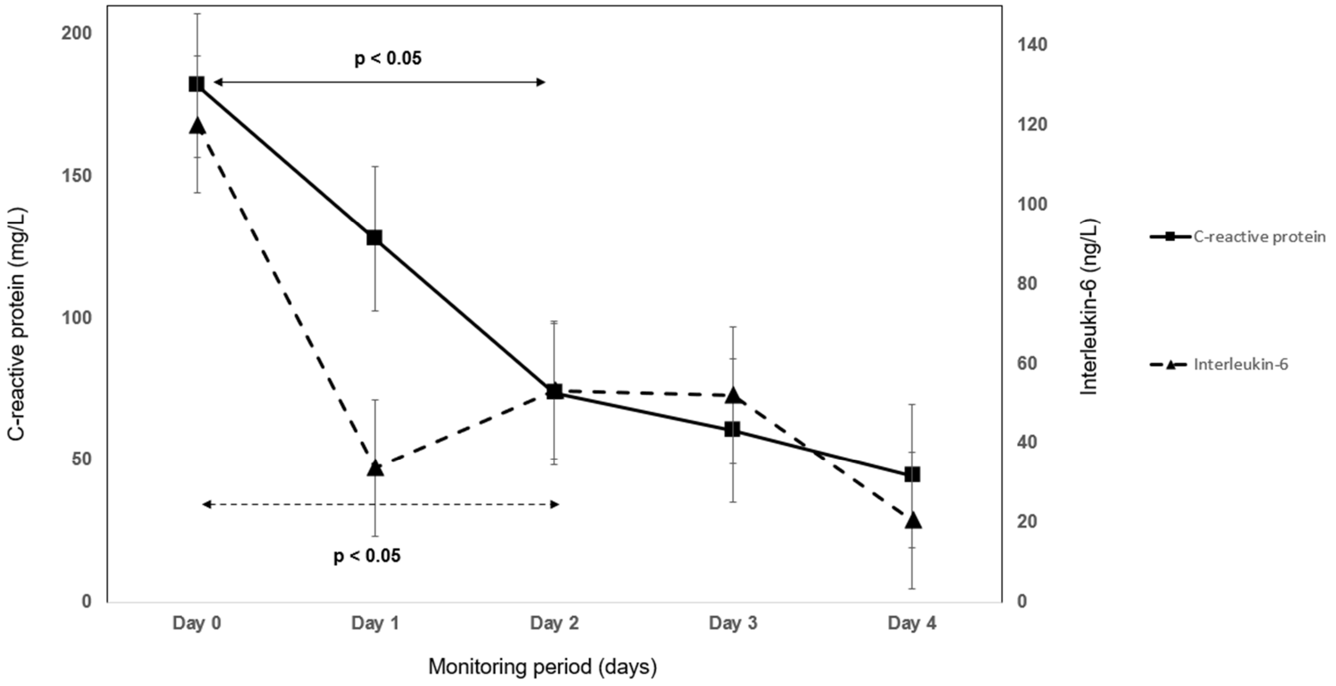

Figure S2: The changes of mean values of C-reactive protein and Interleukin-6 during the monitoring period. P values of the change during the first 48 hours are displayed.

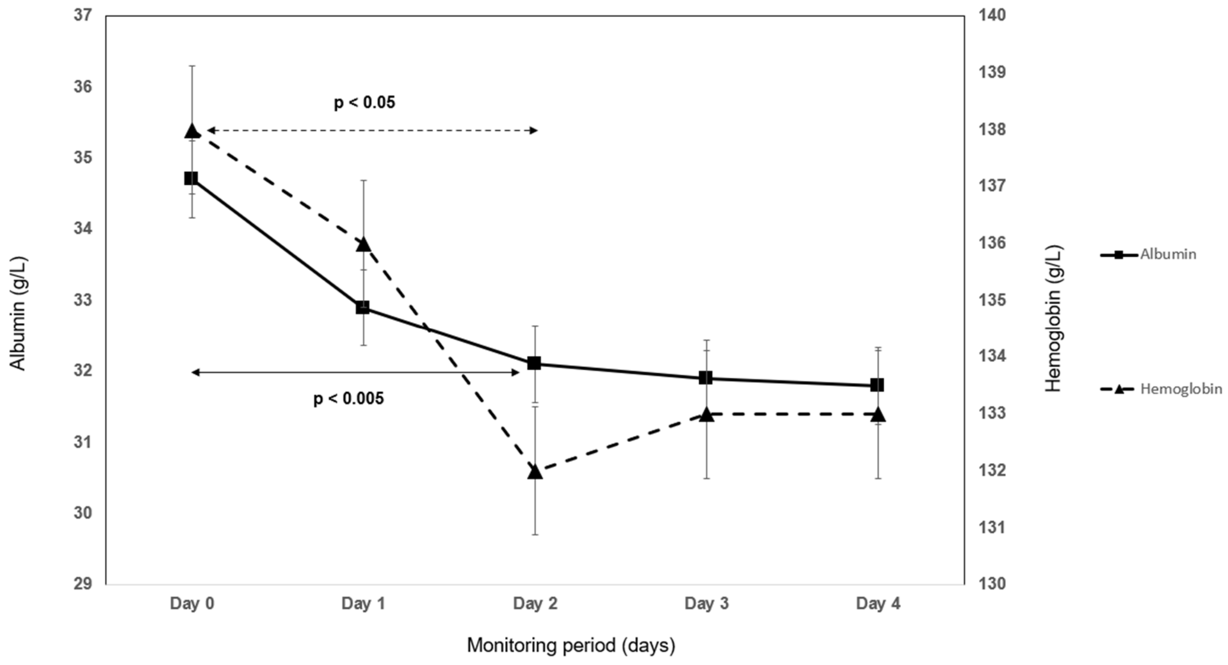

Figure S1: The changes of mean values of albumin and hemoglobin during the monitoring period. P values of the change during the first 48 hours are displayed.
