# Supplementary material for: Serum 25-hydroxyvitamin D Concentration Significantly Decreases in Patients with COVID-19 Pneumonia during the First 48 Hours after Hospital Admission
Source: Nutrients. 2022 Jun 7;14(12):2362. doi: 10.3390/nu14122362 (PMC9228147; doi:10.3390/nu14122362)
Supplement: Supplementary file 1 [file nutrients-14-02362-s001.zip › Nutrients_supplementary_tables.pdf]

Table S1: The mean values of 25(OH)D of each patient during the monitoring period (day 0-4).

Patient	25(OH)D Day 0	25(OH)D Day 1	25(OH)D Day 2	25(OH)D Day 3	25(OH)D Day 4
Pacient 1 (M)	45,20	41,10	39,20	38,00	41,50
Pacient 2 (M)	18,30	15,80	16,60	13,80	15,00
Pacient 3 (M)	39,50	41,30	34,70	36,60	37,60
Pacient 4 (M)	52,30	48,40	45,30	42,00	41,30
Pacient 5 (M)	66,50	50,60	53,70	53,30	56,30
Pacient 6 (M)	30,90	29,30	28,80	26,00	24,90
Pacient 7 (M)	7,15	6,09	8,15	8,92	7,92
Pacient 8 (M)	37,00	36,20	33,20	34,20	30,00
Pacient 9 (M)	11,30	10,40	12,80	8,89	9,42
Pacient 10 (M)	42,40	42,90	38,80	36,70	38,80
Pacient 11 (M)	39,00	37,20	33,30	31,80	31,00
Pacient 12 (M)	30,00	24,00	20,70	25,80	26,90
Pacient 13 (F)	8,94	8,47	7,48	7,11	8,12
Pacient 14 (F)	26,80	20,30	20,10	20,50	21,20
Pacient 15 (F)	21,40	21,80	22,50	23,90	22,90
Pacient 16 (F)	32,70	26,30	25,10	26,00	22,10
Pacient 17 (M)	29,10	31,60	28,50	28,50	31,30
Pacient 18 (M)	5,23	5,38	4,70	5,34	5,21
Pacient 19 (M)	32,30	27,00	26,00	23,00	23,00
Pacient 20 (M)	31,50	27,30	24,20	26,50	25,00
Pacient 21 (F)	47,20	40,20	39,60	36,10	36,90
Pacient 22 (F)	21,00	18,40	16,80	13,90	14,00

Abbreviations: M=male; F=female; 25(OH)D=25-hydroxyvitamin D

Table S2: The mean values of CRP, IL-6, PCT and LD of each patient during the monitoring period (day 0-4).

Patient	CRP Day 0	CRP Day 1	CRP Day 2	CRP Day 3	CRP Day 4	IL-6 Day 0	IL-6 Day 1	IL-6 Day 2	IL-6 Day 3	IL-6 Day 4	PCT Day 0	PCT Day 1	PCT Day 2	PCT Day 3	PCT Day 4	LD Day 0	LD Day 1	LD Day 2	LD Day 3	LD Day 4
**Pacient 1 (M)**	**175,25**	**92,24**	**39,32**	**19,91**	**28,52**	**81,90**	**47,07**	**33,51**	**37,82**	**8,09**	**0,19**	**0,14**	**0,08**	**0,10**	**0,11**	**13,96**	**11,84**	**11,44**	**13,28**	**9,34**
**Pacient 2 (M)**	**107,41**	**59,08**	**25,80**	**15,27**	**8,09**	**77,00**	**1,50**	**2,92**	**7,59**	**3,71**	**0,15**	**0,14**	**0,11**	**0,10**	**0,07**	**9,07**	**7,28**	**6,55**	**6,16**	**5,08**
**Pacient 3 (M)**	**46,38**	**29,94**	**19,92**	**47,51**	**22,68**	**17,89**	**4,53**	**153,60**	**91,92**	**3,21**	**0,06**	**0,05**	**0,06**	**0,04**	**0,02**	**10,33**	**8,34**	**7,95**	**6,80**	**5,54**
**Pacient 4 (M)**	**93,79**	**93,68**	**110,28**	**115,54**	**89,18**	**27,51**	**44,06**	**57,33**	**43,29**	**56,33**	**0,32**	**0,32**	**0,19**	**0,13**	**0,09**	**16,89**	**17,65**	**18,30**	**17,00**	**15,02**
**Pacient 5 (M)**	**198,50**	**163,71**	**87,42**	**46,44**	**68,66**	**172,30**	**8,44**	**12,57**	**6,25**	**43,59**	**0,62**	**0,39**	**0,25**	**0,13**	**0,11**	**17,01**	**14,41**	**13,69**	**9,94**	**10,92**
**Pacient 6 (M)**	**62,07**	**36,30**	**32,38**	**17,22**	**11,07**	**62,22**	**45,82**	**21,28**	**29,06**	**14,34**	**0,24**	**0,14**	**0,10**	**0,05**	**0,06**	**6,86**	**8,08**	**6,70**	**6,40**	**6,49**
**Pacient 7 (M)**	**383,55**	**145,22**	**80,14**	**100,14**	**47,50**	**218,10**	**20,75**	**51,34**	**13,26**	**10,57**	**1,21**	**0,88**	**0,40**	**0,23**	**0,14**	**14,66**	**13,07**	**12,23**	**11,39**	**9,32**
**Pacient 8 (M)**	**193,65**	**207,55**	**118,28**	**54,98**	**26,10**	**188,80**	**67,76**	**8,01**	**3,56**	**4,66**	**0,15**	**0,17**	**0,10**	**0,06**	**0,07**	**6,86**	**7,80**	**6,86**	**6,17**	**5,49**
**Pacient 9 (M)**	**28,07**	**10,61**	**12,12**	**5,38**	**1,96**	**28,65**	**5,82**	**1,50**	**1,50**	**1,50**	**0,12**	**0,09**	**0,09**	**0,09**	**0,08**	**3,69**	**3,16**	**3,15**	**3,50**	**3,26**
**Pacient 10 (M)**	**218,72**	**142,14**	**57,06**	**40,54**	**18,91**	**228,50**	**39,08**	**15,69**	**68,57**	**19,74**	**0,20**	**0,16**	**0,11**	**0,07**	**0,06**	**5,66**	**4,97**	**4,25**	**4,25**	**3,76**
**Pacient 11 (M)**	**207,65**	**94,92**	**51,69**	**60,36**	**30,56**	**58,25**	**36,60**	**39,04**	**23,80**	**11,69**	**0,94**	**0,44**	**0,17**	**0,12**	**0,09**	**6,18**	**5,21**	**5,35**	**5,34**	**4,79**
**Pacient 12 (M)**	**193,82**	**94,21**	**65,23**	**164,04**	**195,25**	**60,87**	**18,43**	**166,10**	**317,40**	**6,43**	**0,19**	**0,09**	**0,08**	**0,18**	**0,29**	**8,43**	**8,75**	**11,97**	**15,84**	**15,41**
**Pacient 13 (F)**	**407,96**	**252,17**	**127,19**	**72,16**	**59,24**	**414,20**	**24,08**	**27,44**	**8,89**	**14,44**	**24,80**	**13,54**	**6,62**	**2,39**	**1,02**	**1,96**	**3,07**	**2,73**	**2,85**	**2,53**
**Pacient 14 (F)**	**91,23**	**84,72**	**131,78**	**174,80**	**98,85**	**17,88**	**87,51**	**80,96**	**44,04**	**2,79**	**0,06**	**0,04**	**0,05**	**0,04**	**0,05**	**6,55**	**5,47**	**7,00**	**7,92**	**6,18**
**Pacient 15 (F)**	**155,55**	**212,39**	**105,44**	**56,62**	**29,31**	**254,90**	**53,79**	**37,07**	**25,00**	**8,18**	**0,36**	**0,42**	**0,32**	**0,51**	**0,41**	**7,47**	**13,70**	**7,12**	**6,30**	**5,91**
**Pacient 16 (F)**	**147,80**	**67,51**	**28,49**	**15,61**	**8,60**	**62,06**	**5,53**	**2,41**	**3,23**	**6,61**	**0,03**	**0,05**	**0,02**	**0,02**	**0,02**	**6,70**	**4,28**	**4,18**	**3,63**	**4,25**
**Pacient 17 (M)**	**128,10**	**70,05**	**67,18**	**38,86**	**17,21**	**62,48**	**72,48**	**2415,00**	**1546,00**	**893,60**	**1,33**	**1,29**	**0,69**	**0,44**	**0,28**	**8,16**	**8,57**	**9,11**	**9,73**	**9,91**
**Pacient 18 (M)**	**170,03**	**133,72**	**44,99**	**21,79**	**13,57**	**62,72**	**12,26**	**8,85**	**9,65**	**4,62**	**10,05**	**9,85**	**4,47**	**2,05**	**1,08**	**3,95**	**6,34**	**5,61**	**7,88**	**5,61**
**Pacient 19 (M)**	**112,51**	**54,92**	**45,20**	**74,43**	**70,39**	**33,57**	**11,79**	**229,40**	**181,50**	**87,36**	**0,39**	**0,21**	**0,23**	**0,26**	**0,26**	**2,34**	**1,81**	**2,54**	**2,45**	**2,42**
**Pacient 20 (M)**	**475,85**	**401,30**	**152,88**	**70,35**	**52,46**	**1130,00**	**265,40**	**64,24**	**34,08**	**170,40**	**1,95**	**1,86**	**0,94**	**0,49**	**0,30**	**12,39**	**16,41**	**12,02**	**12,24**	**12,25**
**Pacient 21 (F)**	**114,47**	**117,42**	**118,66**	**81,39**	**54,42**	**203,40**	**126,40**	**93,02**	**91,94**	**54,19**	**0,21**	**0,15**	**0,11**	**0,10**	**0,09**	**12,80**	**10,54**	**11,28**	**11,41**	**10,53**
**Pacient 22 (F)**	**292,40**	**255,25**	**97,91**	**41,25**	**28,09**	**134,70**	**13,48**	**20,14**	**35,67**	**49,05**	**0,38**	**0,26**	**0,15**	**0,13**	**0,10**	**12,77**	**13,49**	**12,23**	**11,81**	**10,43**

Abbreviations: M=male; F=female; CRP=C-reactive protein; IL-6=Interleukin-6; PCT=Procalcitonin LD=Lactate dehydrogenase

Table S3: The mean values of neutrophils, lymphocytes, monocytes and NLR of each patient during the monitoring period (day 0-4).

Patient	Neutrophils Day 0	Neutrophils Day 1	Neutrophils Day 2	Neutrophils Day 3	Neutrophils Day 4	Lymphocytes Day 0	Lymphocytes Day 1	Lymphocytes Day 2	Lymphocytes Day 3	Lymphocytes Day 4	Monocytes Day 0	Monocytes Day 1	Monocytes Day 2	Monocytes Day 3	Monocytes Day 4	NLR Day 0	NLR Day 1	NLR Day 2	NLR Day 3	NLR Day 4
Pacient 1 (M)	4,04	6,97	7,29	7,35	11,25	1,21	0,90	1,00	1,04	0,80	0,30	0,56	0,67	0,60	0,52	3,00	8,00	7,00	7,00	14,00
Pacient 2 (M)	5,09	8,14	7,46	7,10	7,14	0,84	1,18	1,55	1,77	1,51	0,33	0,72	0,66	0,66	0,65	6,00	7,00	5,00	4,00	5,00
Pacient 3 (M)	2,40	2,57	5,52	5,33	4,63	1,84	1,28	1,92	2,11	2,25	0,39	0,37	0,75	0,74	0,49	1,00	2,00	3,00	3,00	2,00
Pacient 4 (M)	5,33	8,30	10,73	11,27	13,77	1,02	0,88	0,66	0,72	0,64	0,31	0,43	0,49	0,53	0,50	5,00	10,00	18,00	16,00	22,00
Pacient 5 (M)	10,76	4,87	4,07	5,73	7,40	0,76	0,66	0,47	0,66	0,91	0,43	0,55	0,52	0,86	0,95	14,00	7,00	9,00	9,00	9,00
Pacient 6 (M)	7,05	16,03	13,16	11,25	10,08	1,03	1,46	1,29	2,06	2,37	0,41	0,74	0,54	0,71	0,88	7,00	11,00	10,00	5,00	4,00
Pacient 7 (M)	11,19	14,44	13,60	10,96	12,18	0,31	0,33	0,28	0,36	0,50	0,19	0,22	0,35	0,33	0,53	36,00	44,00	49,00	30,00	24,00
Pacient 8 (M)	10,50	15,58	16,46	9,49	8,25	1,25	1,15	1,15	1,60	1,30	0,76	0,72	0,94	0,66	0,64	8,00	14,00	14,00	6,00	6,00
Pacient 9 (M)	3,35	6,32	6,94	4,63	6,17	1,26	1,61	1,55	1,49	1,73	0,15	0,36	0,27	0,23	0,30	3,00	4,00	4,00	3,00	4,00
Pacient 10 (M)	9,22	12,06	11,01	8,20	6,60	0,95	1,05	1,13	1,43	2,07	0,45	0,63	0,66	0,66	0,77	10,00	11,00	10,00	6,00	3,00
Pacient 11 (M)	13,76	12,77	12,71	10,15	9,70	0,94	1,06	0,72	1,10	1,65	0,32	0,80	0,33	0,41	0,47	15,00	12,00	18,00	9,00	6,00
Pacient 12 (M)	1,77	6,98	8,88	11,36	9,03	0,39	0,42	0,57	0,51	0,28	0,13	0,31	0,47	0,44	0,34	5,00	17,00	16,00	22,00	32,00
Pacient 13 (F)	17,73	17,26	13,66	11,73	7,38	0,99	0,85	1,08	2,16	2,38	0,23	0,59	0,54	1,14	0,79	18,00	20,00	13,00	5,00	3,00
Pacient 14 (F)	3,94	4,75	3,59	3,62	5,74	1,75	1,73	2,02	2,23	1,50	0,30	0,34	0,40	0,45	0,53	2,00	3,00	2,00	2,00	4,00
Pacient 15 (F)	5,12	5,55	11,43	7,32	11,59	0,88	0,60	0,60	0,80	0,85	0,83	0,57	1,05	0,57	0,71	6,00	9,00	19,00	9,00	14,00
Pacient 16 (F)	4,97	10,99	7,27	6,92	7,52	0,73	1,44	1,89	1,69	2,14	0,18	0,66	0,57	0,68	0,64	7,00	8,00	4,00	4,00	4,00
Pacient 17 (M)	2,86	6,65	4,99	4,54	6,58	0,27	0,45	0,48	0,54	0,56	0,11	0,24	0,07	0,13	0,21	11,00	15,00	10,00	8,00	12,00
Pacient 18 (M)	13,95	23,54	20,47	16,68	8,74	0,85	0,99	0,45	0,56	0,60	0,36	0,50	0,31	0,52	0,23	16,00	24,00	45,00	30,00	15,00
Pacient 19 (M)	17,02	21,11	16,52	9,48	6,21	0,31	0,65	0,48	0,54	0,91	0,42	0,46	0,44	0,38	0,39	55,00	32,00	34,00	18,00	7,00
Pacient 20 (M)	6,89	6,37	5,35	5,66	7,71	0,45	0,37	0,55	0,59	0,70	0,15	0,16	0,23	0,25	0,23	15,00	9,00	10,00	10,00	11,00
Pacient 21 (F)	6,05	7,08	7,25	9,65	11,19	0,94	1,01	0,67	0,78	0,78	0,51	0,53	0,53	0,74	0,62	3,00	6,00	7,00	11,00	12,00
Pacient 22 (F)	6,00	10,82	9,91	10,78	11,93	0,91	0,94	1,42	1,53	1,94	0,32	0,58	0,54	0,62	0,65	7,00	12,00	7,00	7,00	6,00

Abbreviations: M=male; F=female; NLR=Neutrophil-Lymphocyte Ratio

Table S4: The mean values of albumin, calcium, ionized calcium, hemoglobin and hematocrit of each patient during the monitoring period (day 0-4).

Patient	Albumin Day 0	Albumin Day 1	Albumin Day 2	Albumin Day 3	Albumin Day 4	Clacium Day 0	Calcium Day 1	Calcium Day 2	Calcium Day 3	Calcium Day 4	Ionized Ca Day 0	Ionized Ca Day 1	Ionized Ca Day 2	Ionized Ca Day 3	Ionized Ca Day 4	Hemoglobin Day 0	Hemoglobin Day 1	Hemoglobin Day 2	Hemoglobin Day 3	Hemoglobin Day 4	Hematocrit Day 0	Hematocrit Day 1	Hematocrit Day 2	Hematocrit Day 3	Hematocrit Day 4
Pacient 1 (M)	36,30	32,25	33,60	33,77	32,44	2,31	2,24	2,21	2,22	2,21	1,18	1,19	1,22	1,19	1,16	147,00	145,00	147,00	150,00	152,00	0,45	0,44	0,44	0,45	0,46
Pacient 2 (M)	37,66	37,21	35,04	35,11	38,57	2,24	2,23	2,24	2,29	2,30	1,11	1,20	1,18	1,22	1,21	154,00	149,00	147,00	150,00	152,00	0,47	0,46	0,45	0,47	0,47
Pacient 3 (M)	35,93	36,41	35,69	34,71	35,08	2,14	2,24	2,12	2,19	2,26	1,19	1,19	1,14	1,17	1,23	147,00	149,00	143,00	138,00	147,00	0,45	0,46	0,43	0,42	0,44
Pacient 4 (M)	40,91	39,22	37,63	36,80	36,08	2,18	2,12	2,16	2,17	2,16	1,03	1,12	1,12	1,15	1,13	143,00	138,00	142,00	145,00	139,00	0,44	0,43	0,43	0,44	0,42
Pacient 5 (M)	33,90	32,08	33,62	33,91	33,47	1,95	1,88	2,01	2,04	2,07	1,02	1,05	1,08	1,04	1,09	146,00	139,00	147,00	145,00	144,00	0,43	0,41	0,43	0,43	0,43
Pacient 6 (M)	35,93	39,61	36,67	35,77	33,45	2,02	2,05	2,02	2,15	2,14	1,14	1,12	1,08	1,19	1,15	136,00	136,00	132,00	133,00	129,00	0,41	0,41	0,41	0,41	0,40
Pacient 7 (M)	27,98	26,82	26,49	26,64	25,38	1,92	1,94	1,88	1,83	1,90	1,02	1,02	1,04	1,01	1,06	130,00	127,00	118,00	118,00	119,00	0,39	0,38	0,36	0,36	0,37
Pacient 8 (M)	38,12	36,94	34,75	35,52	32,83	2,09	2,17	2,28	2,22	2,26	1,14	1,14	1,21	1,18	1,24	132,00	148,00	143,00	151,00	149,00	0,40	0,44	0,43	0,45	0,45
Pacient 9 (M)	39,34	37,47	37,20	39,30	39,33	2,17	2,22	2,22	2,23	2,20	1,10	1,14	1,15	1,16	1,08	139,00	140,00	141,00	146,00	148,00	0,42	0,43	0,44	0,45	0,45
Pacient 10 (M)	28,09	27,66	25,08	24,74	25,53	2,17	2,24	2,12	2,13	2,12	1,25	1,25	1,21	1,25	1,21	138,00	142,00	131,00	130,00	131,00	0,42	0,43	0,40	0,40	0,40
Pacient 11 (M)	41,64	34,94	35,68	33,61	31,95	2,30	2,21	2,12	2,21	2,30	1,16	1,18	1,12	1,20	1,07	171,00	154,00	152,00	152,00	155,00	0,52	0,48	0,47	0,47	0,49
Pacient 12 (M)	31,16	28,98	30,87	28,99	28,61	1,96	1,86	1,93	2,00	2,03	1,07	1,10	1,12	1,13	1,25	102,00	93,00	93,00	97,00	94,00	0,32	0,29	0,29	0,31	0,30
Pacient 13 (F)	31,35	31,17	31,00	32,50	33,78	2,03	2,10	2,07	2,08	2,11	1,09	1,18	1,07	1,15	1,14	108,00	102,00	100,00	108,00	103,00	0,33	0,31	0,31	0,33	0,32
Pacient 14 (F)	38,13	31,55	32,02	34,17	32,50	2,17	2,22	2,02	2,13	2,23	1,14	1,13	1,12	1,15	1,16	133,00	133,00	115,00	119,00	120,00	0,41	0,41	0,36	0,37	0,38
Pacient 15 (F)	30,38	33,82	33,55	32,35	34,16	2,27	2,47	2,62	2,59	2,60	1,35	1,35	1,46	1,41	1,49	137,00	152,00	145,00	139,00	136,00	0,42	0,47	0,43	0,43	0,41
Pacient 16 (F)	33,93	34,05	30,87	30,32	31,35	2,22	2,24	2,09	2,17	2,06	1,20	1,22	1,18	1,20	1,14	142,00	137,00	125,00	126,00	128,00	0,44	0,43	0,39	0,40	0,39
Pacient 17 (M)	31,53	30,00	28,80	30,77	31,29	2,02	2,11	2,01	2,19	2,19	1,15	1,15	1,15	1,23	1,26	117,00	118,00	100,00	105,00	106,00	0,36	0,36	0,30	0,32	0,32
Pacient 18 (M)	26,45	29,10	23,50	26,62	24,18	1,88	2,01	1,93	2,04	1,93	1,14	1,16	1,17	1,22	1,13	145,00	153,00	138,00	144,00	139,00	0,45	0,45	0,42	0,43	0,42
Pacient 19 (M)	35,57	29,10	30,21	25,18	24,29	2,68	2,18	2,55	2,22	2,24	1,35	1,30	1,40	1,30	1,33	151,00	137,00	140,00	127,00	122,00	0,45	0,40	0,42	0,38	0,37
Pacient 20 (M)	34,30	29,17	29,76	29,26	29,85	1,96	2,04	2,03	2,07	2,05	1,05	1,09	1,08	1,11	1,09	148,00	135,00	141,00	148,00	150,00	0,44	0,41	0,43	0,45	0,45
Pacient 21 (F)	44,10	35,19	32,50	30,48	31,72	2,20	2,16	2,15	2,20	2,16	1,14	1,14	1,20	1,20	1,19	153,00	140,00	136,00	134,00	139,00	0,47	0,43	0,41	0,41	0,42
Pacient 22 (F)	32,27	30,45	31,43	31,05	33,11	1,96	2,10	2,03	2,03	2,09	0,99	1,09	1,10	1,12	1,14	134,00	138,00	132,00	135,00	141,00	0,42	0,42	0,41	0,42	0,43

Abbreviations:M=male; F=female

Table S5: The mean values of selected kidney and liver markers of each patient during the monitoring period (day 0-4).

Patient	Urea Day 0	Urea Day 1	Urea Day 2	Urea Day 3	Urea Day 4	Creatinine Day 0	Creatinine Day 1	Creatinine Day 2	Creatinine Day 3	Creatinine Day 4	ALT Day 0	ALT Day 1	ALT Day 2	ALT Day 3	ALT Day 4	ALP Day 0	ALP Day 1	ALP Day 2	ALP Day 3	ALP Day 4
Pacient 1 (M)	5,31	7,49	8,15	7,83	7,89	94,30	90,40	78,90	77,90	86,30	0,96	0,94	1,69	4,40	4,63	1,64	1,46	1,82	3,18	2,79
Pacient 2 (M)	4,21	5,87	7,94	7,15	6,67	70,70	70,90	71,60	72,60	66,10	1,62	1,62	1,70	1,89	1,67	1,00	0,96	0,88	0,92	0,98
Pacient 3 (M)	3,58	3,97	5,10	4,93	4,82	74,60	75,40	77,60	78,00	76,10	0,67	1,03	1,90	2,21	2,45	1,05	1,44	1,85	2,18	2,34
Pacient 4 (M)	5,22	6,65	7,62	8,17	7,38	79,70	69,70	67,20	68,50	61,70	6,33	4,25	4,70	2,74	4,25	1,36	1,35	1,47	1,75	1,90
Pacient 5 (M)	3,05	3,94	4,77	4,86	5,32	57,10	50,60	55,80	64,00	71,90	8,61	7,34	9,30	7,85	7,40	2,92	2,59	2,69	2,32	2,26
Pacient 6 (M)	5,37	4,86	5,24	7,07	7,91	73,20	73,30	75,70	81,90	93,20	0,72	1,49	1,10	1,24	2,27	0,83	0,95	0,99	1,15	1,37
Pacient 7 (M)	11,68	18,42	15,62	12,61	10,15	118,90	127,40	97,80	83,10	75,10	1,79	1,26	0,95	0,62	0,59	2,78	2,40	2,10	1,87	1,58
Pacient 8 (M)	2,67	3,17	3,69	4,92	5,49	102,80	87,40	91,10	86,90	87,90	0,63	0,60	0,72	1,24	1,26	0,77	0,81	0,79	0,77	0,74
Pacient 9 (M)	6,59	7,13	7,61	8,62	9,71	91,00	74,90	78,20	80,90	85,60	0,72	0,58	0,53	0,67	0,71	2,40	2,16	2,08	2,07	1,95
Pacient 10 (M)	9,44	10,75	11,60	10,23	9,65	94,40	85,10	85,30	76,80	79,20	0,75	0,85	0,40	1,49	1,59	1,52	1,44	1,18	1,17	1,18
Pacient 11 (M)	22,40	12,38	8,91	8,20	7,18	117,70	77,20	60,20	67,00	61,60	1,30	1,47	1,00	0,97	1,04	3,28	2,49	2,18	2,07	1,93
Pacient 12 (M)	22,80	21,00	14,99	10,77	14,65	265,50	172,30	163,00	143,90	161,00	0,28	0,28	0,28	0,34	0,51	1,16	1,14	1,13	1,24	1,37
Pacient 13 (F)	10,36	8,04	9,64	8,30	6,81	105,20	73,60	83,70	80,10	75,20	0,38	0,74	1,50	1,72	1,07	0,92	1,16	1,35	1,63	1,63
Pacient 14 (F)	6,64	6,29	4,48	3,69	4,56	98,90	78,10	92,00	78,40	82,70	1,90	3,05	3,30	5,27	8,18	4,05	4,73	3,60	5,17	7,31
Pacient 15 (F)	5,54	9,92	17,29	25,97	32,23	78,20	102,40	108,90	146,60	142,70	1,05	4,09	3,09	2,69	2,54	1,49	2,16	2,13	2,40	2,41
Pacient 16 (F)	5,64	6,83	7,47	7,25	5,92	58,70	61,10	66,20	59,90	53,60	0,43	0,63	0,57	0,61	0,96	1,50	1,34	1,17	1,11	1,12
Pacient 17 (M)	13,25	17,79	16,75	16,12	16,53	148,70	155,80	146,50	132,60	137,00	0,39	0,37	0,33	0,36	0,20	3,24	2,77	2,65	2,79	2,70
Pacient 18 (M)	28,15	27,29	19,63	14,32	13,08	193,00	141,20	93,40	75,80	86,90	0,67	0,56	0,56	1,47	1,35	1,21	1,36	1,61	1,63	1,49
Pacient 19 (M)	29,81	23,48	19,89	11,63	8,44	200,60	124,70	115,10	83,60	79,30	0,37	0,29	0,31	0,31	0,37	1,79	1,38	1,56	1,45	1,45
Pacient 20 (M)	10,49	11,80	9,10	9,08	8,77	102,50	74,60	74,40	72,60	79,40	0,41	0,38	0,44	0,57	0,31	1,60	1,44	1,78	1,68	1,47
Pacient 21 (F)	5,82	6,03	7,04	6,27	6,70	73,00	53,80	75,60	60,50	63,80	0,96	0,68	0,81	1,26	0,85	1,50	1,73	1,79	2,18	2,00
Pacient 22 (F)	7,64	7,54	8,79	7,40	7,17	75,00	89,60	89,70	93,70	80,20	0,60	0,51	1,27	2,01	2,24	1,02	1,15	1,26	1,24	1,24

Abbreviations: M=male; F=female; ALT=Alanine transaminase; ALP=Alkaline phosphatase
